# Abnormal Connectional Fingerprint in Schizophrenia: A Novel Network Analysis of Diffusion Tensor Imaging Data

**DOI:** 10.3389/fpsyt.2016.00114

**Published:** 2016-06-30

**Authors:** Sharmili Edwin Thanarajah, Cheol E. Han, Anna Rotarska-Jagiela, Wolf Singer, Ralf Deichmann, Konrad Maurer, Marcus Kaiser, Peter J. Uhlhaas

**Affiliations:** ^1^Department of Neurology, University Hospital of Cologne, Cologne, Germany; ^2^Department of Neurophysiology, Max-Planck Institute for Brain Research, Frankfurt am Main, Germany; ^3^Max-Planck Institute for Metabolism Research, Cologne, Germany; ^4^Department of Electronics and Information Engineering, Korea University, Sejong, South Korea; ^5^Department of Bio-Convergence Engineering, Korea University, Seoul, South Korea; ^6^Department of Brain and Cognitive Sciences, Seoul National University, Seoul, South Korea; ^7^Ernst-Strüngmann Institut, Frankfurt am Main, Germany; ^8^Frankfurt Institute of Advanced Studies, Goethe University Frankfurt am Main, Frankfurt am Main, Germany; ^9^Brain Imaging Centre, Goethe University Frankfurt am Main, Frankfurt am Main, Germany; ^10^Department of Psychiatry, Psychosomatics and Psychotherapy, Goethe University Frankfurt am Main, Frankfurt am Main, Germany; ^11^Interdisciplinary Computing and Complex BioSystems (ICOS) Research, School of Computing Science, Newcastle University, Newcastle, UK; ^12^Institute of Neuroscience, Newcastle University, Newcastle, UK; ^13^Institute of Neuroscience and Psychology, University of Glasgow, Glasgow, UK

**Keywords:** schizophrenia, diffusion tensor imaging, graph theory, connectional fingerprint, neuroinformatics

## Abstract

The graph theoretical analysis of structural magnetic resonance imaging (MRI) data has received a great deal of interest in recent years to characterize the organizational principles of brain networks and their alterations in psychiatric disorders, such as schizophrenia. However, the characterization of networks in clinical populations can be challenging, since the comparison of connectivity between groups is influenced by several factors, such as the overall number of connections and the structural abnormalities of the seed regions. To overcome these limitations, the current study employed the whole-brain analysis of connectional fingerprints in diffusion tensor imaging data obtained at 3 T of chronic schizophrenia patients (*n* = 16) and healthy, age-matched control participants (*n* = 17). Probabilistic tractography was performed to quantify the connectivity of 110 brain areas. The connectional fingerprint of a brain area represents the set of relative connection probabilities to all its target areas and is, hence, less affected by overall white and gray matter changes than absolute connectivity measures. After detecting brain regions with abnormal connectional fingerprints through similarity measures, we tested each of its relative connection probability between groups. We found altered connectional fingerprints in schizophrenia patients consistent with a dysconnectivity syndrome. While the medial frontal gyrus showed only reduced connectivity, the connectional fingerprints of the inferior frontal gyrus and the putamen mainly contained relatively increased connection probabilities to areas in the frontal, limbic, and subcortical areas. These findings are in line with previous studies that reported abnormalities in striatal–frontal circuits in the pathophysiology of schizophrenia, highlighting the potential utility of connectional fingerprints for the analysis of anatomical networks in the disorder.

## Introduction

Recent graph theoretical studies characterize the human brain as a complex network of distinct areas that communicate with each other on different spatial and temporal scales (1). Diffusion tensor imaging (DTI) data and tractography algorithms facilitated the investigation of structural brain networks (2–4) and revealed a brain connectome that is optimized to save connection lengths and maximize communication efficiency (1). In addition, recent studies have highlighted the possibility that psychiatric disorders, such as schizophrenia, are characterized by alterations in the organization of functional and anatomical networks (5–7).

Schizophrenia is a complex psychiatric disorder characterized by positive symptoms, such as hallucinations and delusions, as well as negative symptoms and cognitive dysfunctions. There is emerging evidence that these disturbances are associated with a complex pattern of anatomical and functional connectivity anomalies rather than with impairments of isolated cortical and subcortical structures (8, 9). This is consistent with graph theoretical analyses of anatomical networks that have indicated a widespread disturbance of several local network characteristics, such as clustering coefficient and path length (10–12), while the overall network topology is preserved.

To detect connectivity changes in the network topology in schizophrenia, previous diffusion-weighted imaging studies (13, 14) performed an edge-by-edge comparison of “absolute” sample numbers (absolute connectivity) traced between pairs of brain areas through tractography algorithms and reported reduced cortico-cortical connectivity in several brain regions (12, 13, 15). However, several functional imaging studies reported both hypo- and hyperconnected areas in schizophrenia compared to healthy controls (13). In particular, emerging evidence suggest frontal hyperconnectivity in schizophrenia patients during rest (16, 17) and during working memory (18).

The analysis of absolute connectivity estimates may not be sufficient to characterize aberrant anatomical networks in schizophrenia because they do not account for overall white matter (WM) and gray matter (GM) reductions reported in the disorder (19–21). Hence, we would like to suggest that the analysis of connectional fingerprints in schizophrenia could circumvent some problems associated with absolute connectivity estimates as connectional fingerprints are less affected from the WM and GM reduction than absolute connectivity (22). The connectional fingerprint of a brain area represents the set of connection probabilities to all its target areas (23, 24). Specifically, the connection to each target area is quantified by the number of fiber samples and normalized by the total number of fiber samples propagated from that seed area to all its target areas. Hence, the connectional fingerprint provides a unique set of connection probabilities for each brain region (23) that is independent of overall WM reduction and differences in the seed areas (22). Recently, the connectional fingerprint was employed for analyses of functional connectivity (25).

Recent diffusion tensor imaging studies in healthy participants facilitated the investigation of connectional fingerprints to distinguish functionally different subunits in the thalamus (24), in the medial frontal cortex (26), in the geniculate bodies (27), in the lateral frontal cortex (28, 29), and in the lateral prefrontal cortex of humans (30) and macaques (22). Brain regions defined through their connectional fingerprint corresponded to parcelations based on anatomical characteristics, including patterns of gyri and sulci (30) and localization based on functional magnetic resonance imaging (fMRI) data, suggesting a possible link between the connectional fingerprint and the function of a brain area (29, 31).

In the current study, we employed connectional fingerprints to provide a novel insight into organizational network changes in schizophrenia. The analysis of connectional fingerprint has so far been applied only to specific brain areas (24, 26–29, 31) and only in healthy participants (32, 33). Thus, to our knowledge, the current study is the first whole-brain investigation of connectional fingerprint in a clinical sample. Specifically, we hypothesized that connectional fingerprints in frontal and subcortical areas will be abnormal in the line with the notion of a disconnection syndrome (34). To examine this question, we first detected brain regions whose overall connectional fingerprint differed between groups through similarity analysis and then tested for correlations with clinical symptoms.

## Materials and Methods

### Participants

We recruited 16 medicated schizophrenia patients who met the DSM-IV criteria for schizophrenia from the Department of Psychiatry, Johann Wolfgang Goethe University, Frankfurt. We also recruited 17 age- and gender-matched healthy controls from the local community. All patients were on atypical neuroleptics at the time of testing. DSM-IV diagnosis for schizophrenia was confirmed by a trained psychologist with the SCID-interview for DSM-IV-R. Exclusion criteria were for both schizophrenia patients and controls: (1) a neurological disorder, (2) alcohol, nicotine, or substance dependence within the last month, or (3) structural abnormalities in the T1 MR image. After being given a complete description of the study, each participant provided written informed consent. The study was approved by the ethical committee of the Goethe University Frankfurt. Participants were assessed with the Edinburgh Handedness Inventory (35), the Brief Assessment of Cognition in Schizophrenia (BACS) battery (36), and the Mehrfach-Wortwahl-Test (37), which is a measure of premorbid, verbal intelligence. Current psychopathology of schizophrenia patients was assessed with the Positive and Negative Syndrome Scale (PANSS) (38) and symptoms were grouped into five factors according to the model of Lindenmayer et al. (39), including the components “positive,” “negative,” “cognitive,” “excitement,” and “depression.” In addition, we assessed patients for the “disorganization factor” that comprises the items conceptual disorganization, poor attention, and inappropriate affect (40).

### Data Acquisition

MR-data were acquired on a 3-T scanner (Siemens Trio, Erlangen, Germany) at the Brain Imaging Centre (BIC), Frankfurt am Main, Germany. The imaging protocol included high-resolution T1-weighted anatomical MRI and diffusion tensor MRI. The scanner’s body coil was used for RF transmission and an eight-channel head coil for signal reception. Head motion was minimized by the use of tightly padded clamps. The T1-weighted anatomical imaging protocol had the following parameters: three-dimensional (3D) MPRAGE, sagittal slices, field of view (FoV) = 256 mm × 256 mm × 176mm, matrix size 256 × 256 × 176, isotropic resolution of 1 mm, TR/TE/TI = 2250 ms/2.6 ms/900 ms, FA = 9°, bandwidth = 200 Hz/pixel. The DTI protocol was chosen as follows: 2D slice selective spin echo EPI sequence with diffusion encoding, FoV = 192 mm × 192mm, matrix size 96 × 96, 60 axial slices with a thickness of 2 mm, no interslice gap, isotropic resolution of 2 mm, TR/TE = 8200 ms/99 ms, FA = 90°, bandwidth = 1302 Hz/pixel, echo spacing = 0.85 ms. For DTI analysis, 60 diffusion encoding directions with a *b*-value of 1000 s/mm^2^ were covered, and 10 baseline images without diffusion weighting were acquired. We recorded DTI three times for each subject to improve the signal-to-noise ratio with a scanning time of 36 min.

### Data Preprocessing

A brain network consists of nodes that are predefined anatomical regions and edges, which reflect the relationships between any pair of two nodes. To define the nodes, we employed the Harvard-Oxford Probabilistic MRI atlas developed by FMRIB Oxford and the Harvard Centre for Morphometric Analysis that was predefined on the MNI template based on anatomical landmarks (http://www.cma.mgh.harvard.edu/fsl_atlas.html). We included 48 cortical and 7 selected subcortical areas (thalamus, nucleus caudatus, putamen, pallidum, amygdala, nucleus accumbens, and hippocampus) for each hemisphere (Table S1 in Supplementary Material for the list of nodes and their abbreviation). Using FSL tools from the FMRIB software library (41), we skull-stripped DTI-images without diffusion encoding and a T1-weighted image (42, 43). For each participant, we obtained coregistration between these two images, and non-linear registration between a skull-stripped T1-weighted image and the MNI template, using FNIRT (42). Furthermore, we transformed the selected regions of interest defined on the MNI template into the diffusion-weighted images of individuals to ROIs for probabilistic tracking. We note that we visually checked all procedures of skull-stripping, coregistration between each T1 image and its corresponding DTI, and registration between the Harvard-Oxford atlas and diffusion-weighted images.

**Figure 1:**
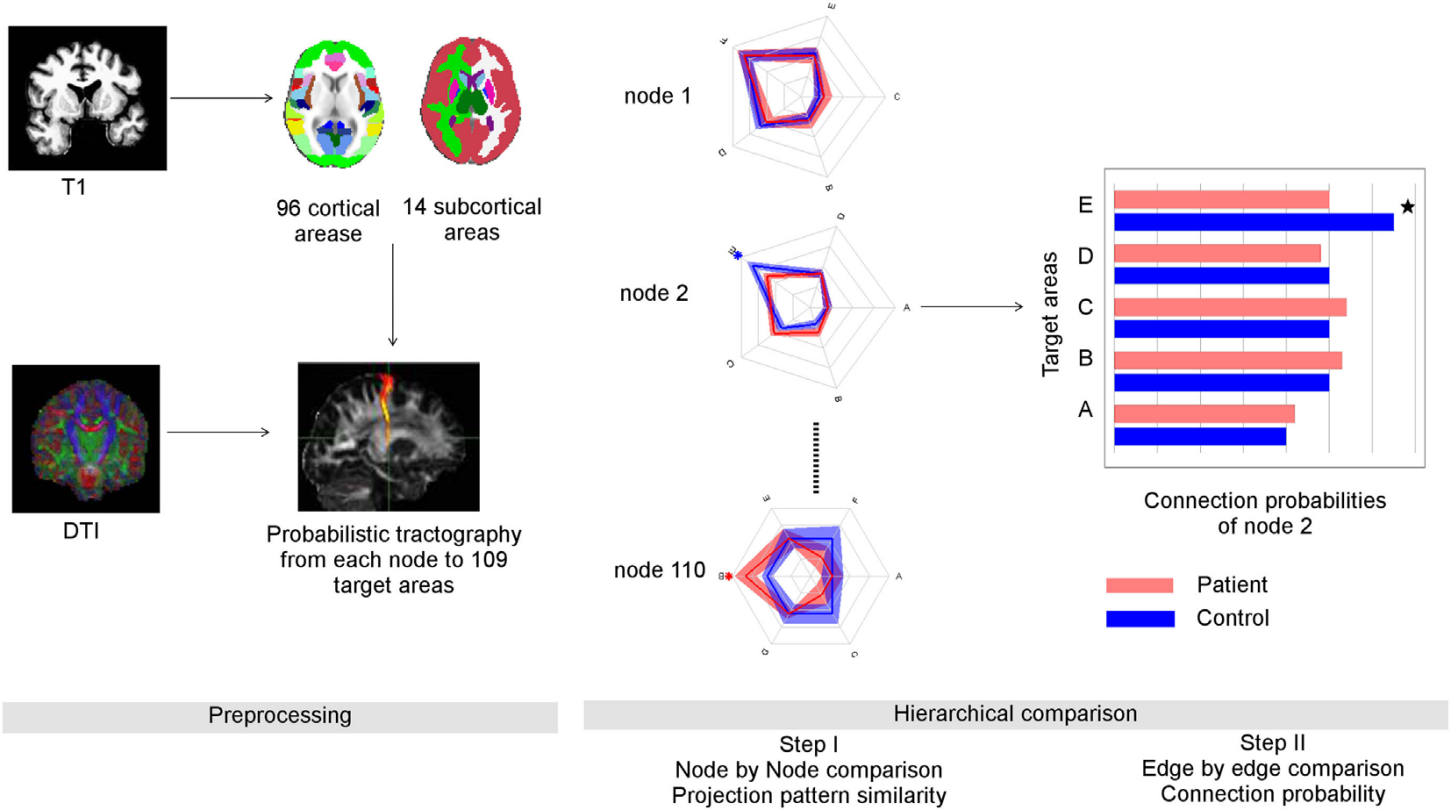
**Overview of data processing and suggested graph theoretical analysis**. Left panel: data preprocessing: anatomical data was parcellated into 96 cortical and 14 subcortical regions using FSL (Harvard-Oxford probabilistic atlas, Table S1 in Supplementary Material). Through probabilistic tractography, the fiber samples were detected voxel-wise from each seed area to the 109 target areas. Right panel: the number of fiber samples that reached a certain target area B from seed area A divided by all fiber samples propagated from A provided the connection probability between A and B. The whole set of connection probabilities of seed area A provided its connectional fingerprint that was visualized as a radial graph. Here, we show the simplified finger prints of three nodes with five target areas each. The distance from the center represented the connection probability. After comparing the similarity of connectional fingerprints node by node, we performed *post hoc* tests to identify abnormal connection probabilities that lead abnormal connectional fingerprints. In this example, the connection probability of node two to its target area E was reduced in schizophrenia patients compared to controls.

To quantify edges, we performed probabilistic tractography (44). Probabilistic tractography has several advantages over deterministic tractography (45), in particular in the detection of tracts among crossing fibers (46) as well as the reconstruction of fibers also in areas of low anisotropy (47). Moreover, its test–retest reliability is robust (48, 49). Before further processing the diffusion tensor images, we serialized three diffusion tensor images and gradient vectors for each subject; thus, a collated image of each subject has 30 b0-images and 180 diffusion images (50). Then, we corrected effects of head motion and eddy currents, registering images with diffusion encodings to the image without diffusion encoding of the first scan; we adequately rotated the gradient vectors after the correction. Subsequently, we performed local modeling of probabilistic diffusion parameters (bedpostX) (44) over the skull-stripped diffusion-weighted images. Then, we conducted probabilistic tracking in DTI space using the classification target tool in ProbtrackX2 (FSL) with 5000 samples/voxel and 110 registered ROIs based on the Harvard-Oxford atlas as explained above. The overall pipeline, including preprocessing, was shown in Figure 1.

### Network Construction and Connectional Fingerprint

Each brain region has the unique set of connection probabilities to all its target areas, which is called its “connectional fingerprint” (23). The connectional fingerprint of each brain area is defined by its target areas and the connection probabilities to these target areas (44). With probabilistic tractography, we detected the number of fiber samples of each seed voxel that reached each of the 109 target areas. The number of fiber samples that reached a certain target area divided by the total number of samples propagated from a voxel represented the connection probability of the seed voxel to a specific target area. We extended this procedure to all voxels in the seed area and averaged their connection probability. Hence, the connectional fingerprint of the node *i* (*w^i^*) includes the connection probability to a node *j* that, was defined as:

$$w_{j}^{i}=\frac{1}{n^{i}}\sum_{q}\frac{c_{jq}^{i}}{\sum_{k}c_{kq}^{i}}$$

where $c_{jq}^{i}$ represents the number of fiber samples from a voxel *q* of the node *i* to the target node *j*, and *n^i^* represents the number of seed voxels in the node *i*. The connection probability of the voxel *q*$\left(c_{jq}^{i}\text{/}\Sigma_{k}c_{kq}^{i}\right)$ was averaged for all voxels in the node *i*.

It is important to note that the connectional fingerprint is a node-specific characteristic. Since it is acquired through nodal normalization, $w_{j}^{i}$ is not equal to $w_{i}^{j}$. $w_{j}^{i}$ is the connection probability of node *i* that takes into account all connections from node *i*, while $w_{i}^{j}$ is normalized for all connections from node *j*. As a simple example, when the total number of fiber samples propagated from the node *j* is bigger than the one from the node *i*, connection probability $w_{i}^{j}$ would be smaller than $w_{j}^{i}$ (see text 1 in Supplementary Material).

### Identifying Brain Regions with Abnormal Connectional Fingerprint

We identified nodes with abnormal connectional fingerprints through permutation testing of pairwise similarity between any two participants for a certain node. We computed the sum of the absolute difference between two connectional fingerprints and transformed it to a similarity measure using a simple monotonically decreasing exponential function. The similarity of the *k*th node between the *i*th subject and the *j*th subject is:

$$s_{ij}^{k}={\text{ e}}^{-}{\;}^{d_{ij}^{k}/\alpha }\text{ where }d_{ij}^{k}=\left|w_{i}^{k}-w_{j}^{k}\right|$$

where $w_{i}^{k}$ is the connectional fingerprint of the *i*th subject from the *k*th node, |⋅| represents a sum of all elements’ absolute value, and α is a regularization factor, which was set to the half of the maximum difference over all nodes and subjects.

For each node, we computed the pairwise similarity of the corresponding connectional fingerprints for all subjects in both groups (*n* × *m* similarity matrix, where *n* is the total number of subjects and *m* is the number of nodes) and the significance level defining a group difference was obtained through permutation testing under the assumption that the average within-group similarity is larger than the average between-group similarity (51) for a connectional fingerprint that differs significantly between groups. To estimate the significance level, we constructed a null distribution of the representative statistic with permutations of group assignment, where the representative statistic is the difference between average within-group similarity and average between-group similarity. Specifically, we computed the value for *N* − 1 randomly permuted group assignments, where the significance level was estimated as the number of values, which is equal to or larger than the value from the original group assignment divided by *N*, where *N* is the number of permutations. A multiple comparison correction for nodes was obtained through the false discovery rate (FDR) procedure (52). We note that this method can be applied to multi-group comparison. We evaluated the suggested statistical test over synthetic data in the Supplementary Material (See SI text 2, Figures S1, S2, and Table S2 in Supplementary Material).

### *Post hoc* Tests

To identify the connection probabilities that led to the between-group differences in the connectional fingerprints, we then compared the connection probabilities of the corresponding fingerprints between groups using permutation testing (12, 53) and FDR. Since the connection probabilities of the connectional fingerprint of a certain node are independent from that of another node, multiple comparison correction can be performed separately for each identified node. We note that the connection probability between two areas has to be analyzed in the context of the whole connectional fingerprint of a node.

### Correlation with Clinical Symptoms

Pearson correlation coefficients were computed between the PANSS-factors (positive, negative, cognitive, depression, excitement, and disorganization), individual PANSS-items and connection probabilities for those nodes with abnormal connectional fingerprints.

### Statistics and Visualization

All topological and statistical operations were conducted using Matlab (version 2009b, Mathworks, Natick, MA, USA). Statistical testing was performed with a permutation test (50,000 permutations) (53) and FDR procedure (54) for multiple comparison correction with a threshold of *q* = 0.05. A permutation test was employed because the connectivity data were not normally distributed. We implemented the proposed method using Matlab, and uploaded both the codes and the test data set at http://cheolhan.dothome.co.kr/software/.

For visualization routines, we used our in-house code, which draws brain regions and connection probabilities overlapped with the transparent brain. Coordinates of each region were retrieved from the FSL package by calculating the center of mass. As the connectional fingerprint is a node-specific parameter, we used radial graphs (23) to visualize the distribution of each brain region’s connectional fingerprint or bar graphs. In a radial graph, the distance represented the magnitude of each connection probability from the graph’s center.

## Results

### Participants

We recruited 16 medicated schizophrenia patients [mean age: 34.2 6 ± 9.80 SD, age range: (21–52)] and 17 age- and gender-matched healthy controls [mean age: 32.31 ± 9.79, age range: (19, 51)]. All patients were on medication at the time of testing receiving atypical antipsychotics. Both groups did not differ significantly in age (permutation test, *p* = 0.82), gender (*p* = 0.60), handedness (*p* = 0.81), and years of education (*p* = 0.41). There was a statistical trend for a difference in verbal intelligence (*p* = 0.06) between groups. Compared to controls, schizophrenia patients achieved lower values in the total score of the BACS battery (*p* = 0.00001) and in the following subtests: verbal memory (*p* = 0.0004), digit sequencing (*p* = 0.0001), verbal fluency (*p* = 0.00001), and symbol coding (*p* = 0.0044) (Table 1).

**Table 1:** **Demographic data**.

	Controls (*N* = 17)	Patients (*N* = 16)	*p*-values*
Gender	Male = 10	Male = 11	0.60
Age	32.3 ± 9.8^a^	34.3 ± 9.8	0.82
Education (years)	15.0 ± 3.8	13.9 ± 3.0	0.41
Verbal IQ	29.9 ± 2.8	28.9 ± 3.4	0.06
Handedness	68.8 ± 25.8	70.5 ± 23.4	0.81
**PANSS**			
Positive	–	11.7 ± 4.6	–
Negative	–	16.9 ± 6.8	–
Cognitive	–	11.4 ± 4.0	–
Excitement	–	5.6 ± 1.7	–
Depressive	–	13.6 ± 3.3	–
Total Score	–	68.3 ± 17.7	–
**BACS**			
Verbal memory	54.5 ± 5.9	40.19 ± 13.5	**0.0004**
Digit Sequencing	25.9 ± 3.3	20.06 ± 4.6	**0.00001**
Motor speed	77.7 ± 32.2	68.94 ± 22.9	0.2088
Verbal fluency	60.9 ± 12.5	40.12 ± 15.5	**0.00001**
Symbol coding	19.7 ± 5.9	12.47 ± 5.3	**0.0044**
Reasoning and problem solving	58.5 ± 11.3	47.71 ± 12.4	0.0872
Total score	421.1 ± 55.4	326.69 ± 69.1	**0.00001**

*^a^Mean ± SD*.

*Significant values are shown in bold.

### Abnormal Connectional Fingerprints in Schizophrenia

**Figure 2:**
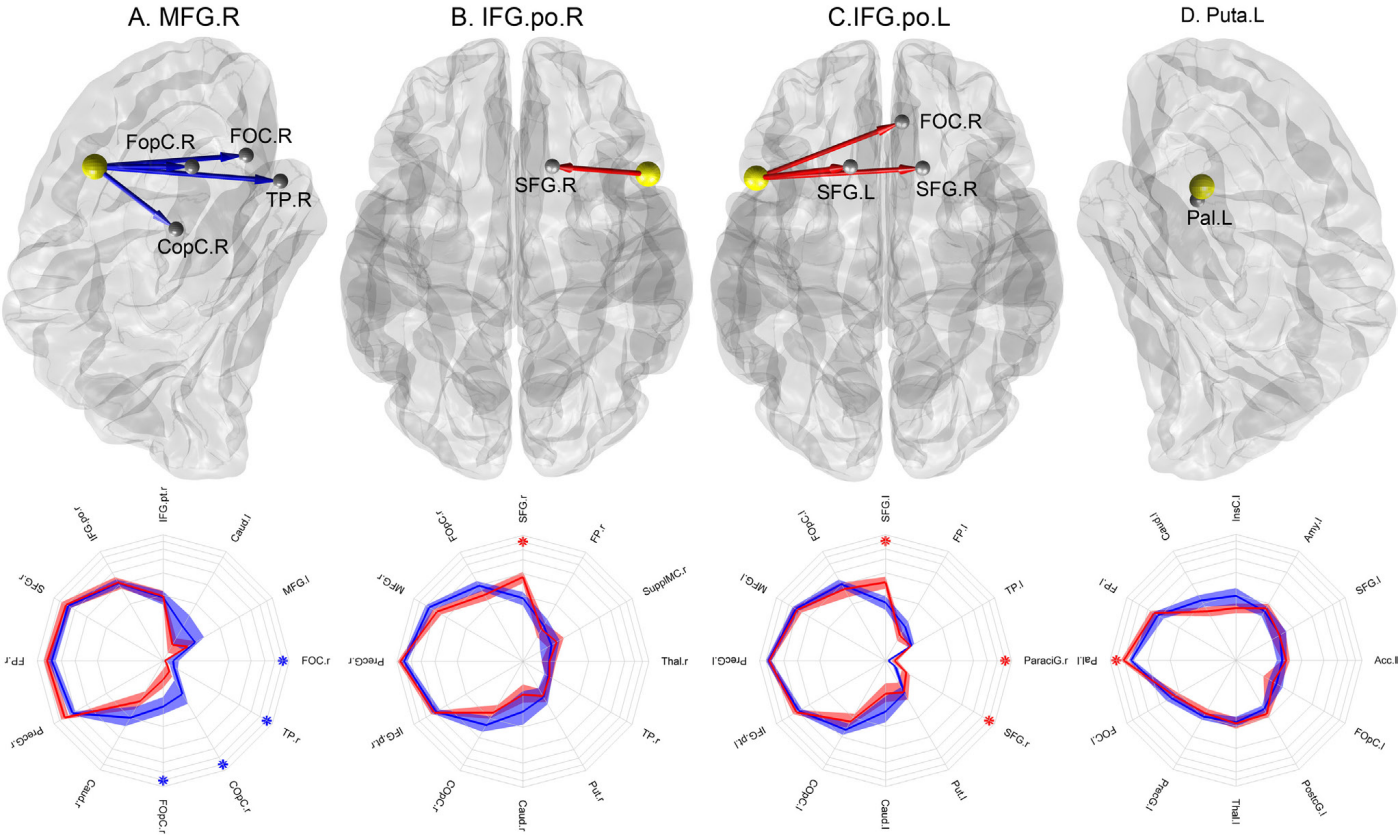
**Nodes with abnormal connectional fingerprints in schizophrenia**. Top row: **(A)** MFG.r in a lateral view of the right hemisphere, **(B)** IFO.po.r in a transverse view, **(C)** IFO.po.l in a transverse view, and **(D)** Puta.l in a lateral view of the left hemisphere. The yellow spheres represent the nodes with abnormal projections and the gray spheres represent their target areas. The blue arrows indicate significantly reduced connection probabilities, while the red arrows indicate significantly increased connection probabilities in schizophrenia patients compared to controls. Bottom row: the corresponding connectional fingerprint diagram for each node with abnormal connection probabilities presented with selected normal connection probabilities. The direction represents different target areas, while the distance from the center represents the magnitude of the connection probability in the log-scale: each concentric circle represents 0.05, 0.1, 0.15, 0.2, 0.25, and 0.3 in order. The blue lines represent average connection probabilities in control subjects, and the blue shades capture their 95% confidence intervals. The red lines represent average connection probabilities in patients and red shades capture their 95% confidence intervals. The blue asterisks indicate significantly increased connection probabilities in schizophrenia patients, while the red asterisks indicate significantly increased connection probabilities in controls. We visualized the top 12 strongest connection probabilities in IFG.po.r and Puta.l; however, in case of MFG.r and IFG.po.l, the significant results were not among them, we included the significant results first.

Four abnormal nodes were identified in schizophrenia patients: right medial frontal gyrus (MFG), left putamen (Puta) and the opercular part of the left and right inferior frontal gyrus (IFG.po) (Figure 2) (FDR-corrected, *p* < 0.05).

To identify the abnormal connection probabilities within each connectional fingerprint, we performed *post hoc* tests (Table 2). The significance level of connection probabilities using connectional fingerprint diagrams is illustrated in Figure 2 and Figures S3–S6 in Supplementary Material. As summarized in Table 2, except for findings in the connectional fingerprints of MFG.r, all the other significantly different connection probabilities were higher in schizophrenia patients compared to controls relative to the other connection probabilities in the corresponding connectional fingerprints. In the connectional fingerprints of MFG.r, we found reduced connection probabilities to the right Frontal Operculum Cortex (FOpC.r), the right Frontal Orbital Cortex (FOC.r), the right Temporal Pole (TP.r), and the right Central Opercular Cortex (COpC.r). In the connectional fingerprint of IFG.po.r, the connection probability to the right superior frontal gyrus (SFG.r) was increased in schizophrenia patients compared to controls. In the connectional fingerprint of IFG.po.l, we found increased connection probabilities to both the superior frontal gyrus (SFG) and the right paracingulate gyrus (ParaciG.r). In the connectional fingerprint of Puta.l, schizophrenia patients showed a higher connection probability to the right pallidum (Pal.r).

**Table 2:** ***Post hoc* tests of abnormal connectional fingerprints**.

Nodes with abnormal connectional fingerprints	Target area^a^	Pseudo*t*-statistics^b^	Significance level (FDR adjusted)
MFG.r	FOpC.r	3.2388	*p* = 0.0391
	FOC.r	2.1950	*p* = 0.0391
	TP.r	2.7085	*p* = 0.0391
	COpC.r	2.4573	*p* = 0.0391
IFG.po.r	SFG.r	**−3.6284**	*p* = 0.0066
IFG.po.l	SFG.l	**−3.4542**	*p* = 0.0110
	SFG.r	**−3.2642**	*p* = 0.0110
	ParaciG.r	**−2.9178**	*p* = 0.0117
Puta.l	Pal.l	**−3.0792**	*p* = 0.0484

*Permutation test, FDR adjusted. See Table S1 in Supplementary Material for abbreviations*.

*^a^Target area of abnormal connection probability within the abnormal connectional fingerprint*.

*^b^Controls’ mean–patients’ mean, normalized by mean and SD over permutations, thus, negative pseudo *t*-statistics represents increased connection in patients (shown in *bold*)*.

### Correlation of Abnormal Projections with Clinical Symptoms

We calculated the Pearson correlation between PANSS-factors and the connection probabilities that showed significant group differences. While no significant correlations between the main factors of the PANSS and the connection probabilities were detected, we found the following significant correlations with individual PANSS-items: in the connectional fingerprint of the MFG.r, the connection probability with COpC.r was correlated with “lack of spontaneity” (*p* = 0.0048, *r* = 0.7062), “blunted affect” (*p* = 0.0133, *r* = 0.6420) and ‘‘poor attention’’, the connection probability with FOC.r was correlated with “depression” (*p* = 0.0414, *r* = −0.5503). In the connectional fingerprint of the Puta.r, the connection probability with Pal.r correlated with “disorientation” (*p* = 0.0407, *r* = 0.5520). In the connectional fingerprint of the IFG.po.r, the connection probability with SFG.r correlated with “disorientation” (*p* = 0.0202, *r* = 0.6111) and “depression” (*p* = 0.0107, *r* = 0.6569). These results did not survive multiple comparison correction, however. No significant correlation with BACS scores was observed.

## Discussion

### Abnormal Connectional Fingerprints in Schizophrenia

Our study provides the first whole-brain analysis of connectional fingerprints in a clinical sample. Through connectional fingerprint analysis of DTI-data, we provide a complementary view of connectivity changes in schizophrenia that is consistent with the “dysconnection” hypothesis (34). The dysconnection hypothesis suggests that schizophrenia is not only characterized by reduced connectivity but also involves hyperconnected areas in several networks, such as the cortico-subcortical pathways (55, 56), the default-mode network (18, 57), and the language processing network (58, 59).

Our data suggest connectivity abnormalities in several nodes of the frontal cortex, a brain region that has been traditionally implicated in the pathophysiology of schizophrenia (60). Specifically, our findings in the MFG and the inferior frontal gyrus (IFG) are consistent with graph theoretical studies that have indicated abnormal organization and connectivity of frontal networks (10, 11). Functional studies suggest that schizophrenia patients show hyperconnectivity of frontal areas during rest (18). In addition, aberrant modulation of frontal activity has been implicated in a number of cognitive dysfunctions in schizophrenia, including working memory (61) as well as impaired social communication (62, 63) and self-other distinction (64).

A second brain region characterized by abnormal connectional fingerprints was the putamen. Striatal circuits and their interactions with cortical regions, in particular with frontal areas, are crucially involved in working memory (65) as well as in reward processing during normal brain functioning (66). Functional and anatomical studies in schizophrenia have provided evidence for impaired striatal–cortical processes underlying these processes (67–69), suggesting that abnormal, striatal connectional fingerprints could contribute toward behavioral abnormalities in schizophrenia. This hypothesis is furthermore supported by the fact that in the connectional fingerprint of the MFG the connection probabilities to the left and right Caudate showed reduced values in schizophrenia patients albeit an uncorrected significance level (see Figure S1 in Supplementary Material).

Possible reasons for connectivity anomalies in schizophrenia may be aberrant myelination (70) and pruning during different developmental stages (71) that impair normal brain maturation with segregation of anatomically close areas and integration of distributed functionally specialized areas (72). In addition, changes in connectivity may also be due to the prolonged presence of positive symptoms that could lead to changes in the architecture of the cortical network as a result of use-dependent strengthening of cortico-cortical connections (73).

### Methodological Issues

Investigations into the organization of anatomical networks remain challenging in clinical populations because of abnormalities in GM/WM-volume that can confound streamline-numbers detected through tractography algorithms, for example, as these are influenced by overall WM connectivity, and the volume of the seed area in GM. Accordingly, edge comparison analysis based on the number of streamlines or fiber tracts (absolute connectivity) might not be sufficient.

The use of the connectional fingerprint has several potential advantages: first, connection probabilities of connectional fingerprints are independent from the overall connectivity (22), because they are normalized for all fibers of the seed area. Second, connectional fingerprints are also independent from the seed volume and, respectively, the total brain volume (33), because each pattern is first calculated for each voxel and subsequently averaged for the whole seed area. Hence, we suggest that a connectional fingerprint is suitable to investigate the “local” or node-specific connectivity pattern more precisely (33, 74) compared to approaches involving the normalization of connectivity between each pair of nodes by the total number of fiber tracts (75).

However, our methodological approach has a several limitations that need to be considered in future studies. First, it is important to note that the selection of the similarity measure determines the sensitivity of the tests and alternative measures have to be studied in future studies (See text 3 in Supplementary Material). Second, probabilistic tractography is sensitive to the distance bias. Hence, if there were systematic differences in the distances between two brain regions in patients compared to healthy subjects, the connectional fingerprints would differ between groups. Thus, our result should be carefully interpreted.

It is important to note that significant difference in the connectional fingerprints between groups is detected if either few connection probabilities within a connectional fingerprint differ between groups by a large magnitude or many connectional probabilities within a connectional fingerprint differ by a small magnitude. This was also confirmed by the analysis of synthetic data (See text 2 in Supplementary Material). For the *post hoc* tests of MFG.r and IFG.po, we observed significant differences in very small mean connection probabilities. However, small connection probabilities may result from crossing fibers and long distances between seed and target areas. Hence, changes in the tail of the connectional fingerprint need to be interpreted with care. However, it should be emphasized that connection probabilities that showed relative hyperconnectivity in patients only involved paths with strong mean connection probabilities in the connectional fingerprint (from IFG.po.r to SFG.r; from IFG.po.l to SFG.l, and from Puta.l to Pal.l, See Figure 2).

Another limitation of our study is the relatively small sample size. However, our results of abnormal connectional fingerprints (Figure 2) and connection probabilities (Figures S3–S6 in Supplementary Material) were significant after the multiple comparison correction. In addition, we would like to note that we did not acquire EPI distortion field maps and did not perform EPI distortion field correction. Thus, the registration can be inaccurate and we may have inaccurate tractography results in regions with high EPI distortion. However, we visually validated the registration to account for this. In addition, we cannot exclude the possibility that some of the changes observed in connectional fingerprints are due to effects associated with antipsychotic-medication treatment. In particular, abnormalities observed in the putamen could be influenced by antipsychotic medication as this region rich in D2 receptors that are targets for antipsychotic medication (76).

## Conclusion

In summary, we present a new approach to systematically investigate local connectivity independent of overall GM/WM changes of seed areas through connectional fingerprint analysis. This approach provides novel evidence for an altered organization of the connectome in schizophrenia that implicates changes in the network architecture of WM pathways as a core anatomical deficit. Specifically, we demonstrate altered connectional fingerprints in frontal and subcortical areas involving both hypo- and hyperconnectivity relatively to the other connection probabilities in their corresponding connectional fingerprints. Future studies need to address the functional consequences of abnormal connectional fingerprints. Since our findings are in line with previous graph theoretical analysis of fMRI data (13), we suggest that connectional fingerprint analysis is a useful parameter approach in multimodal studies that investigate the link between functional and structural data. This hypothesis is strengthened by previous studies that demonstrated high correspondence between parcelation based on the connectional fingerprints and segregation based on task-specific fMRI data (29, 31).

## Author Contributions

SET, CEH, MK, PJU designed the research and wrote the manuscript. SET collected the data. SET, CEH, ARJ preprocessed the data. SET, CEH developed the method and analyzed the data. WS, RD, KM contributed towards manuscript preparation with interpretation.

## Conflict of Interest Statement

The authors declare that the research was conducted in the absence of any commercial or financial relationships that could be construed as a potential conflict of interest.

## Abbreviations

Acc, nucleus accumbens; Amy, amygdala; Caud, nucleus caudatus; COpC, central opercular cortex; FOC, frontal orbital cortex; FopC, frontal operculum cortex; FP, frontal pole; IFG.po, inferior frontal gyrus pars opercularis; IFG.pt, inferior frontal gyrus, pars trigonum; InsC, insular cortex; MFG, medial frontal gyrus; Pal, pallidum; ParaciG, paracingulate gyrus; PostcG, Postcentral Gyrus; PrecG, precentral gyrus; Put, putamen; SFG, superior frontal gyrus; SupplMC, supplementary motor cortex; Thal, thalamus; TP, temporal pole, and r and l denoted the right hemisphere and the left hemisphere, respectively (see Table S1 in Supplementary Material).
