# Supplementary material for: Abnormal Connectional Fingerprint in Schizophrenia: A Novel Network Analysis of Diffusion Tensor Imaging Data
Source: Front Psychiatry. 2016 Jun 30;7:114. doi: 10.3389/fpsyt.2016.00114 (PMC4928135; doi:10.3389/fpsyt.2016.00114)
Supplement: Supplementary file 1 [file data_sheet_1.pdf]

Supplementary Material

Abnormal connectional fingerprints in Schizophrenia: A Novel Network Analysis of Diffusion Tensor Imaging Data

(Sharmili Edwin Tharanarajah; Cheol E. Han; Anna Rotarska-Jagiela; Wolf Singer; Ralf Deichmann; Konrad Maurer; Marcus Kaiser; Peter J. Uhlhaas)

Table S1. Abbreviation of brain areas

Index	Abbreviation	Brain Region
1	FP	Frontal Pole
2	InsC	Insular Cortex
3	SFG	Superior Frontal Gyrus
4	MFG	Medial Frontal Gyrus
5	IFG.pt	Inferior Frontal Gyrus, pars trigonum
6	IFG.po	Inferior Frontal Gyrus,pars orbitale
7	PrecG	Precentral Gyrus
8	TP	Temporal Pole
9	STG.ad	Superior Temporal Gyrus, anterior division
10	STG.pd	Superior Temporal Gyrus, posterior division
11	MTG.ad	Medial Temporal Gyrus, anterior division
12	MTG.pd	Medial Temporal Gyrus, posterior division
13	MTG.top	Medial Temporal Gyrus, temperooccipital part
14	ITG.ad	Inferior Temporal Gyrus, anterior division
15	TG	Temporal Gyrus
16	ITG	Inferior Temporal Gyrus, temporal occipital part
17	PostcG	Postcentral Gyrus
18	SPL	Superior Parietal Lobule
19	SupramG.ad	Supramarginal Gyrus, anterior division
20	SupramG.pd	Supramarginal Gyrus, posterior division
21	AngG	Angular Gyrus
22	LOC.sd	Lateral Orbital Cortex, superior division
23	LOC.id	Lateral Orbital Cortex, inferior division
24	IcalcC	Inferior Calcarine Cortex
25	FMC	Fronto Medial Cortex
26	SupplMC	Supplementary Motor Cortex
27	SubcalcC	Subcalcarine Cortex
28	ParaciG	Paracingulate Gyrus
29	CiG.ad	Cingulate Gyrus, anterior division
30	CiG.pd	Cingulate Gyrus, posterior division
31	PrecunC	Precuneal Cortex
32	CunC	Cuneal Cortex
33	FOC	Frontal Orbital Cortex

34	ParahG.ad	Parahippocampal Gyrus, anterior division
35	ParahG.pd	Parahippocampal Gyrus, posterior division
36	LingG	Lingual Gyrus
37	TFC.ad	Temporal Fusiform Cortex, anterior division
38	TFC.pd	Temporal Fusiform Cortex, posterior division
39	TOFC	Temporo Occipital Fusiform Cortex
40	OFG	Occipital Fusiform Cortex
41	FOpC	Frontal Operculum Cortex
42	COpC	Central Opercular Cortex
43	Pop	Parietal Operculum Cortex
44	PlPol	Planum Polare
45	HG	Heschl's Gyrus
46	PT	Planum Temporale
47	SupracC	Supracalcarine Cortex
48	OP	Occipital Pole
49	Thal	Thalamus
50	Caud	Nucleus caudatus
51	Puta	Putamen
52	Pal	Pallidum
53	Amy	Amygdala
54	Acc	Nucleus Accumbens
55	Hipp	Hippocampus

SI Text 1. Difference between absolute connect ivy and connectional fingerprints

Two connection probabilities w_j^i and w_i^j are different because they are parts of different connectional fingerprints of the nodes i and j. The connectional fingerprint captures the connection probability relative to the sum of all connections starting from the respective node. Hence w_j^i can have a higher value than w_i^j, if the connection probability w_i^j is not a major connection of the node j but w_j^i is a main connection in the connectional fingerprint of the node i. For an example, the connection probability between the anterior division of the right middle temporal gyrus (MTG.ad) and its posterior division (MTG.pd) showed a significant difference regarding the connectional fingerprints of MTG.ad and MTG.pd (paired *t*-test; t =21.89, p<0.001; MTG.ad to MTG.pd: 0.29±0.06; MTG.pd to MTG.ad: 0.11±0.03) but absolute connectivity did not significantly differ between them (paired *t*-test; t =-1.32, p=0.20; MTG.ad to MTG.pd: 240.97±53.75; MTG.pd to MTG.ad: 250.39±59.27). This is because the connection was the largest among edges from MTG.ad but not among edges from MTG.pd. MTG.pd has more connections than MTG.ad (MTG.ad: 978.88±193.50, MTG.pd: 2376.87±464.28). Thus, the connection was about 25 % of the all connections from MTG.ad, while it is about a tenth of the all connections from MTG.pd. Consequently, the relative importance of the connection is higher in the connectional fingerprints of MTG.ad than in the connectional fingerprints of MTG.pd.

Thus, the connectional fingerprint obtained from probabilistic tractography is asymmetric. Since DTI itself cannot assess the directionality of fibres, previous studies including Gong et al. (2009) symmetrized the connection probability ($S_{\overline{ij}}$) between two nodes i and j through averaging as follows:

$$S_{\overline{ij}} = (w_j^i + w_i^j)/2$$

where w_j^i is the connection probability between i and j in the projection pattern of node i, and w_i^j is the connection probability between i and j in the connectional fingerprint of node j. Gong et al. (2009) justified this procedure by pointing out that the correlation between w_j^i and w_i^j was high in their dataset. In agreement with this data, the correlation between w_j^i and w_i^j for all participants in the current study was similarly high (r=0.82 ± 0.0121). However, since two connection probabilities w_j^i and w_i^j are conditional probabilities derived from a unique connection distribution of the node i and the node j in order, they cannot be averaged as shown above. Thus, we suggest that the connectional fingerprints should not be symmetrized as proposed by Gong et al. (2009).

In contrast, 'absolute connectivity' can be symmetrized. The 'absolute connectivity' from node i to node j (a_j^i) is defined by the number of samples propagated from node i, that reached node j:

$$a_j^i = \sum_q c_{jq}^i$$

where c_{jq}^i represents the number of fibre samples from a voxel *q* of the node *i* to the target node j using the nomenclature of the main text. Although a_j^i is slightly different from a_i^j due to the tractography algorithm, their correlation was very high (r=0.93±0.0131), and significantly higher than the correlation between w_j^i and w_i^j (paired *t*-test; t=-41.16, p<0.001). Following Gong et al (2009), the higher correlation may strengthen our assumption that absolute connectivity is more suitable to be symmetrized than the connection probabilities provided in the connectional fingerprints.

SI Text 2. Evaluating permutation testing of similarity for connectional fingerprints with synthetic data

We tested the suggested approach over synthetic data to evaluate its performance. First, since connection probabilities of a connectional fingerprint sums up to one, the basis connectional fingerprint is necessary, which represents a healthy subject. For simplicity, it has 100 connection probabilities and thus 0.01 for each connection probabilities. Second, we added a small contrast to a randomly selected connection probability with a magnitude of 10% of the selected connection probability (i.e. 0.001) which representing a patient. Third, we replicated the synthetic healthy subjects and patients 50 times each, and added a zero-mean Gaussian noise which forms inter-subject variability. When its standard deviation is σ, the contrast-to-noise ratio (CNR) is simply 0.001/ σ. After adding the noise, we re-normalized each connectional fingerprint to make the summation of its connection probabilities one. Afterwards, we performed statistical tests: 1) the suggested approach, and 2) permutation testing of each connection probabilities between groups with the false discovery rate (FDR) procedure (Benjamini and Hochberg 1995) for the multiple comparison correction; the second simple test is significant when any connection probability showed a significant difference after the FDR procedure. Next, we repeated the last two steps 100 times to construct receiver operating characteristic (ROC) curves and compute area-under-curves (AUC). The ROC curve plots the true positive rate (TPR) against the false positive rate (FPR), capturing the sensitivity and (1-specificity) in order. If the curve approaches the top-left corner, the test has very good sensitivity and specificity. Similarly, as the AUC value is larger, the performance of the test is better.

There are two possible conditions that lead to significant differences in the connectional fingerprints between groups with the suggested approach: a) few connection probabilities that differ between groups by a large magnitude and b) many connectional probabilities that differ by a small

magnitude. In our study, the connection probabilities of Puta.l differ by a large magnitude between groups, while the MFG.R show many connection probabilities with small differences between schizophrenia patients and controls. So, we repeated the evaluation above for various numbers of contrasts. We tested the condition that 1, 3, 5, 7, and 10 connectional probabilities have contrasts along with three CNR conditions: 0.5, 1, and 1.5. When the number of contrast is small and CNR is high, it is the case of a), while it is the case of b) when the number of contrast is large and CNR is low since low CNR means small magnitude of contrasts. We collected AUC values for all cases (Table S2, Figure S1) and visualized the ROC curve in the range of the false positive rate (FPR) between 0.001 and 0.1 since FPR represents α-level of the test (Figure S2).

When the number of contrasts is one and CNR is high (Figure S2), the permutation test with the FDR procedure performs better than the suggested approach. However, the purpose of this study is not to detect a single very significant connection probability but to detect overall differences in the connectional fingerprint. Our approach is especially sensitive in the condition of low CNR (Figure S2); it helps to detect the overall difference in the connectional fingerprint. Moreover, since the AUC values were quite similar between two tests (Figure S1, Table S2), our suggested approach showed comparable performance with the permutation tests with the FDR procedure when there is a large peak in the connectional fingerprint while it showed excellent performance otherwise.

Another advantage of the suggested approach is that it provides the smaller p-values than the other approach. To perform the whole brain analysis of the connectional fingerprints, the multiple comparison correction over brain regions is also required. Thus, the smaller p-value of each brain region will ease the multiple comparison correction over brain regions. However, p-values from the permutation test with the FDR procedure are already inflated. For examples, where the number of contrasts equals to one and CNR equals to 1.5, though the AUC values is slightly higher in the permutation test with the FDR procedure, the mean p-value over 100 runs was smaller in the suggested approach (suggested approach: 0.0002 vs. the permutation test with FDR: 0.0066).

Table S2. Area-Under-Curve (AUC) values for synthetic data

number of contrasts	CNR†	Suggested approach	Permutation tests w/ FDR
1	0.5	0.572	0.470
	1.0	0.820	0.961
	1.5	0.955	0.975
3	0.5	0.735	0.743
	1.0	0.991	0.988
	1.5	0.995	0.995
5	0.5	0.848	0.863
	1.0	0.995	0.994
	1.5	0.995	0.995
7	0.5	0.945	0.903
	1.0	0.995	0.995
	1.5	0.995	0.995
10	0.5	0.959	0.929
	1.0	0.995	0.995
	1.5	0.995	0.995

†Contrast-to-Noise Ratio

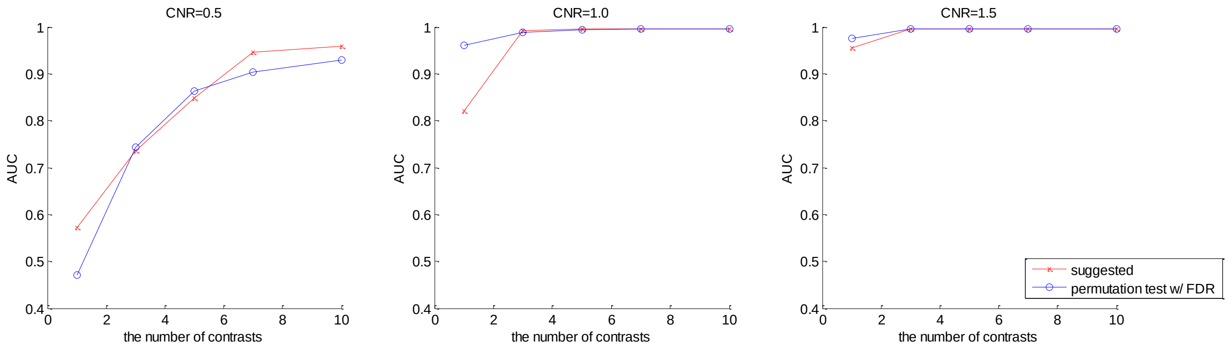

Figure S1. Area-under-curve (AUC) values for synthetic data with respect to the number of contrasts and contrast-to-noise ratio (CNR). The AUC values from Table S2.

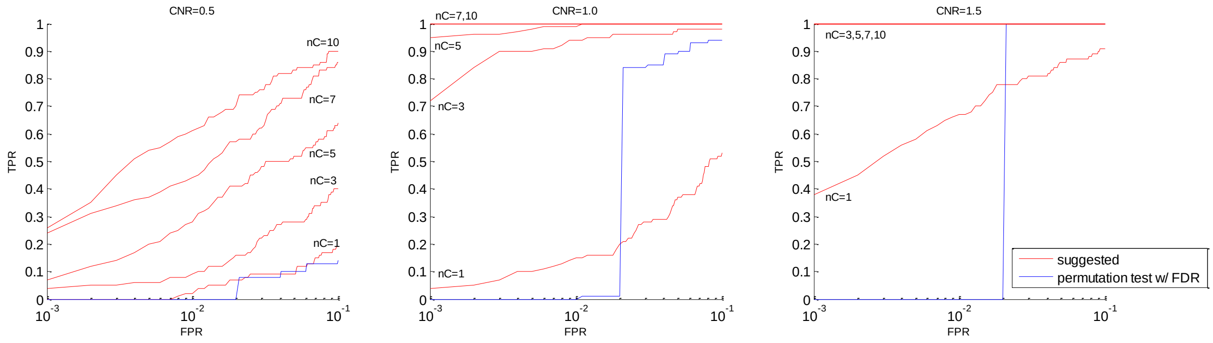

Figure S2. Receiver operating characteristic (ROC) curves in the range of FPR between 0.001 and 0.1 for synthetic data with respect to the number of contrasts (nC) and contrast-to-noise ratio (CNR).

SI Text 3. Similarity measures of connectional fingerprints

In our proposed approach, the definition of the similarity measure was crucial. We computed the sum of absolute difference between the connection probabilities of the corresponding connectional fingerprint and transformed it to a similarity measure using a simple monotonically decreasing exponential function. We tested two alternative similarity measures: a cosine similarity and a simple Pearson correlation. The cosine similarity, $s_{ij}^{k} = p_k^i \cdot p_k^j / \left| p_k^i \right| \times \left| p_k^j \right|$ assesses the angle between two vectors in the 109-dimensional space, which represent the connectional fingerprints. The Pearson correlation between two vectors assesses how strongly they vary together.

Compared to what we suggested in the manuscript, these similarity measures were less sensitive to identify the local changes in the connectional fingerprint. Using the cosine similarity, only the connectional fingerprint of the medial frontal gyrus (MFG.r) was different between groups. The Pearson correlation approach identified both inferior frontal gyrus, pars opercularis (IFG.po.r and IFG.po.l). These alternative similarity measures show lower sensitivity, because they capture the overall difference in general. The cosine similarity evaluates the overall difference in terms of the angle between two vectors in the high dimensional space, which could be too small to be sensed since the vectors resided in the very high dimensional space. Similarly, the Pearson correlation captures the overall degree that two vectors co-vary. The similarity measure we used in the manuscript regarding the sum of all absolute errors of each connectional probability captures also the local differences. In addition, we noted that the shape of the exponential function might help to detect even small differences.

Reference

Benjamini, Y. and Y. Hochberg (1995). "Controlling the false discovery rate: a practical and powerful approach to multiple testing." Journal of the Royal Statistical Society. Series B (Methodological): 289-300.

Gong, G., P. Rosa-Neto, F. Carbonell, Z. J. Chen, Y. He and A. C. Evans (2009). "Age- and gender-related differences in the cortical anatomical network." J Neurosci **29**(50): 15684-15693.

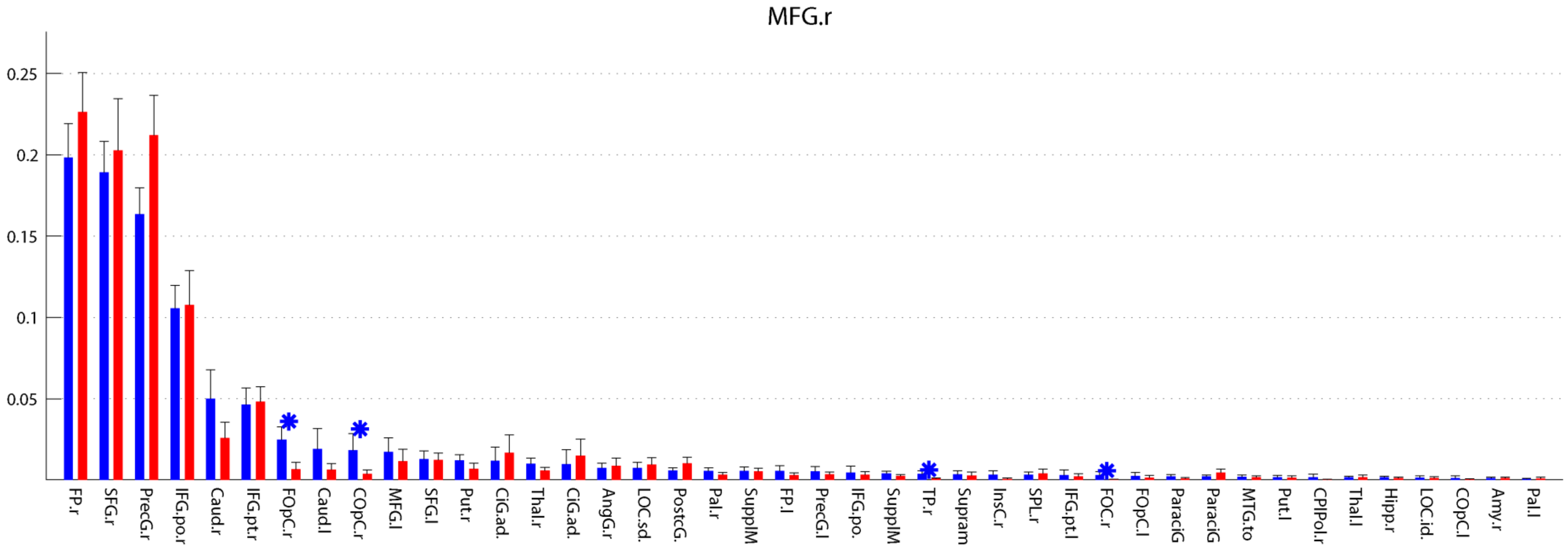

Figure S3. Abnormal connection probabilities from the right medial frontal gyrus (MFG.r). Blue bars represent the controls and red bars represent the patients, where asterisks showed the significantly different connection probability. We showed the connection probabilities whose mean values in the healthy controls equal to or are above 0.001. The abbreviation of regions can be found in Table S1

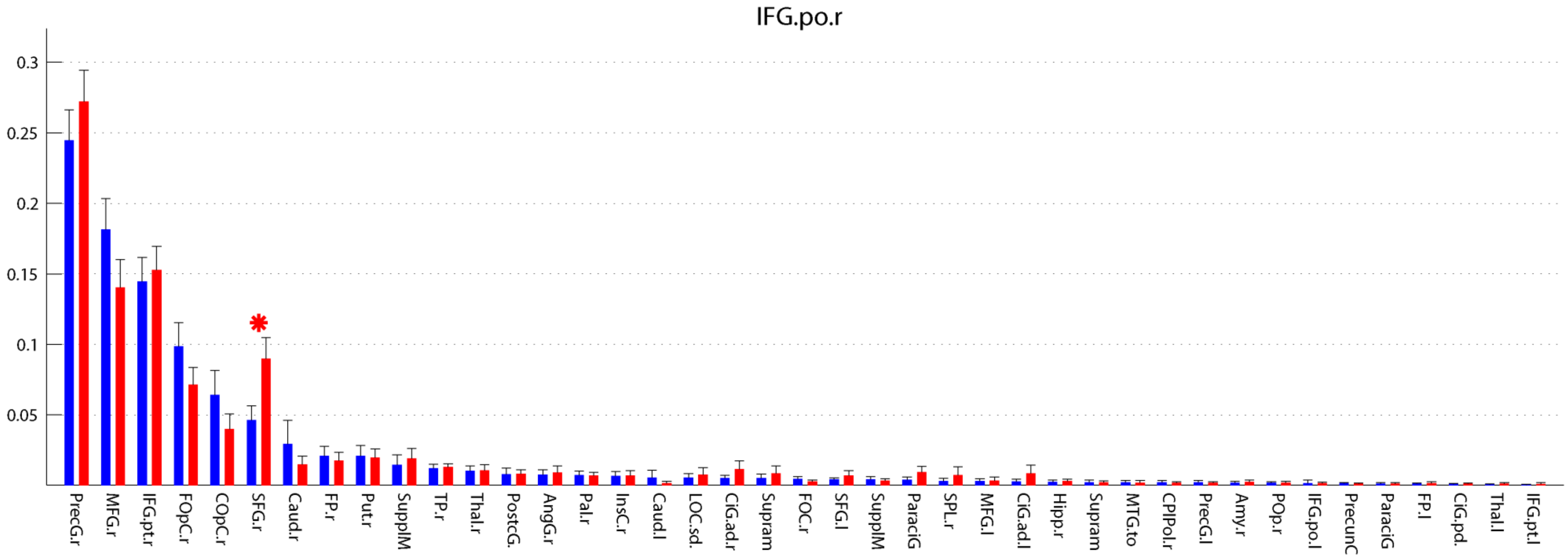

Figure S4. Abnormal connection probabilities from the right inferior frontal gyrus, pars opercularis (IFG.po.r). Blue bars represent the controls and red bars represent the patients, where asterisks showed the significantly different connection probability. We showed the connection probabilities whose mean values in the healthy controls equal to or are above 0.001. The abbreviation of regions can be found in Table S1

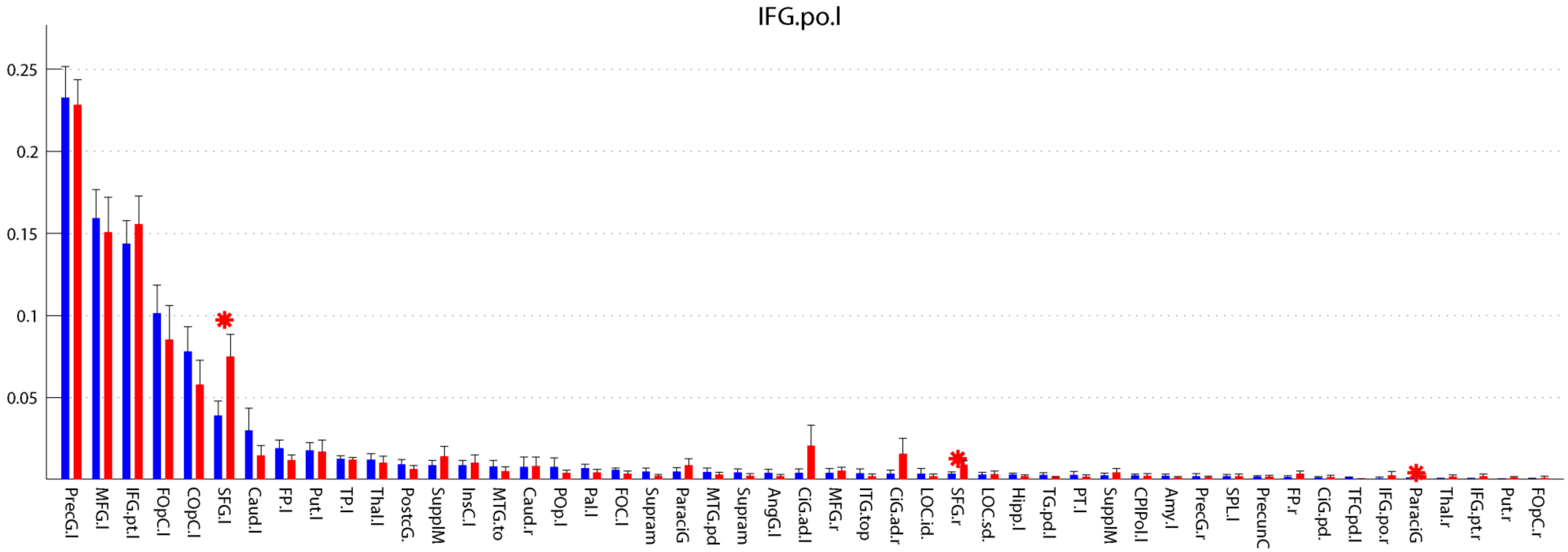

Figure S5. Abnormal connection probabilities from the left inferior frontal gyrus, pars opercularis (IFG.po.l). Blue bars represent the controls and red bars represent the patients, where asterisks showed the significantly different connection probability. We showed the connection probabilities whose mean values in the healthy controls equal to or are above 0.001. The abbreviation of regions can be found in Table S1

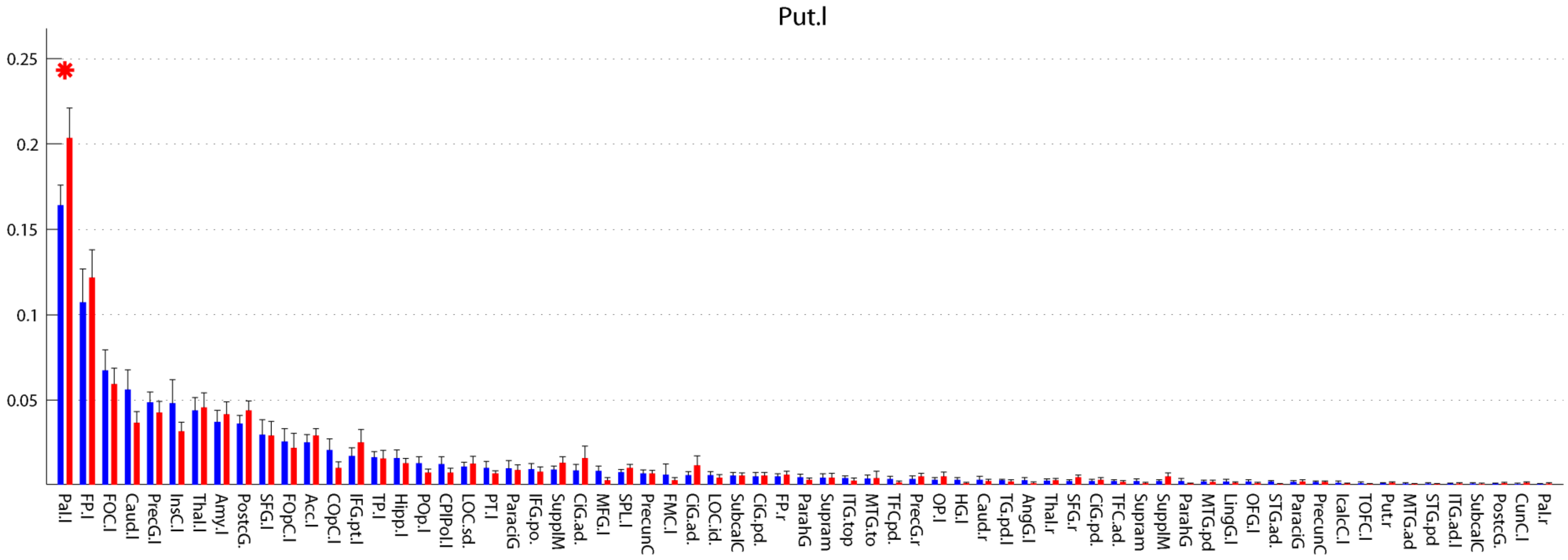

Figure S6. Abnormal connection probabilities from the left putamen. Blue bars represent the controls and red bars represent the patients, where asterisks showed the significantly different connection probability. We showed the connection probabilities whose mean values in the healthy controls equal to or are above 0.001. The abbreviation of regions can be found in Table S1.
